# Risk assessment of personal exposure to polycyclic aromatic hydrocarbons and aldehydes in three commercial cooking workplaces

**DOI:** 10.1038/s41598-018-38082-5

**Published:** 2019-02-07

**Authors:** Ming-Tsang Wu, Pei-Chen Lin, Chih-Hong Pan, Chiung-Yu Peng

**Affiliations:** 1 Department of Public Health, College of Health Sciences, Kaohsiung Medical University, 100 Shih-Chuan 1st Rd, Kaohsiung, Taiwan; 2 Research Center for Environmental Medicine, Kaohsiung Medical University, 100 Shih-Chuan 1st Rd, Kaohsiung, Taiwan; 3 Department of Family Medicine, Kaohsiung Medical University Hospital, Kaohsiung Medical University, 100, Tzyou 1st Road, Kaohsiung, Taiwan; 4 Graduate Institute of Clinical Medicine, College of Medicine, Kaohsiung Medical University, 100 Shih-Chuan 1st Rd, Kaohsiung, Taiwan; 5 Research Center for Cijin Cohort, Kaohsiung Medical University, 100 Shih-Chuan 1st Rd., Kaohsiung, Taiwan; 6 Institute of Labor, Occupational Safety and Health, Ministry of Labor, No. 99, Ln. 407, Hengke Rd., Sijhih District, New Taipei City, Taiwan; 7 Department of Medical Research, Kaohsiung Medical University Hospital, Kaohsiung Medical University, 100, Tzyou 1st Road, Kaohsiung, Taiwan

## Abstract

Cooking-related emissions are associated with environmental pollution and adverse health effects. Of the various chemical species emitted during cooking, polycyclic aromatic hydrocarbons (PAHs) and aldehydes are two chemical species with carcinogenic or tumor promoting characteristics. Although PAH exposure has been studied in commercial kitchen workers, few studies have investigated simultaneous exposure to PAHs and aldehydes in these workers. The aims of this study were to compare personal concentrations of PAH and aldehyde in three commercial cooking workplaces and to estimate their corresponding cancer risks. The three cooking workplaces included western fast food restaurant kitchens, Chinese cafeteria kitchens, and street food carts. Comparisons showed that workers in western fast food restaurant kitchens and Chinese cafeteria kitchens tended to have lower personal concentrations of these pollutants compared to workers in street food carts. The geometric mean (95% CI) cancer risks in the three workplaces were, from lowest to highest, 1.36 (1.12–1.67) × 10^−5^ for western fast food restaurant kitchens, 1.52 (1.01–2.28) × 10^−5^ for Chinese cafeteria kitchens, and 3.14 (2.45–4.01) × 10^−5^ for street food carts. The percentage contributions of aldehyde species to cancer risk were very high (74.9–99.7%). Street food cart workers had high personal exposure to aldehyde probably due to lack of effective exhaust systems. Thus, their cancer risk was significantly higher than those of workers in western fast food restaurant kitchens (p < 0.001) and Chinese cafeteria kitchens (p = 0.013).

## Introduction

Cooking-related emissions are a public health concern for several reasons. First, cooking activities produce harmful pollutants^1–3^ such as polycyclic aromatic hydrocarbons (PAHs), aldehydes, volatile organic compounds, ultrafine particles (particles smaller than 100 nm in diameter), and PM2.5 (particulate matters smaller than 2.5 μm in diameter). Second, epidemiological studies performed in China, Taiwan, Hong Kong, and Singapore have linked cooking oil fume (COF) exposure to lung cancer in nonsmoking women^4,5^. Notably, the International Agency of Research on Cancer has also categorized emissions from high-temperature frying as a probable carcinogen (Group 2A)^6^. Thus, cooking emissions have potentially adverse impacts on human health.

Of all pollutants emitted by cooking activity, the two most frequently investigated species are PAHs and aldehydes. The PAHs have attracted the interest of researchers because they are generated at the high temperatures used for cooking^7^ and because of their well-recognized carcinogenicity^8^. However, most studies of PAHs have only analyzed area concentrations in family/commercial kitchens and in exhaust air^9,10^. Personal exposure to PAHs is rarely reported^11–13^. Notable findings in the literature on PAH include the following: PAH levels increase with cooking temperature^14^, the cooking method that generates the most PAHs is barbecue cooking^10^, and particulate PAHs are a larger contributor to benzo(a)pyrene equivalent (BaP_eq_) concentrations compared to gaseous PAHs^9^.

Aldehydes are produced by degradation of fatty acids. Aldehyde emissions are associated with cooking temperature^15,16^ and with the fatty acid composition of oil used for cooking^17,18^. Whereas formaldehyde and acetaldehyde are known and probable carcinogens, respectively^6^, high carbon number aldehydes (e.g., t,t-2,4-nonadienal, t,t-2,4-decadienal (t,t-2,4-DDE)) are known mutagens with tumor promoting characteristics^19–21^.

Professional cooks have high potential risk of exposure to cooking-related emissions such as PAHs and aldehydes^2^. Few studies have investigated simultaneous occupational exposure to these two species. The objective of this study was to investigate occupational exposure to PAHs and aldehydes and their corresponding cancer risks.

## Results

### Area air concentrations

The geometric mean (GM) concentration of total PAH was substantially higher in the street food cart group (8790.2 ng/m^3^) compared to the Chinese cafeteria kitchen and western fast food restaurant kitchen groups (3721.1 and 3171.0 ng/m^3^, respectively). Most PAHs were 2-ring or 3-ring PAHs, which have lower toxic equivalent factors compared to other PAHs. The most potent carcinogen, benzo(a)pyrene, was only detected in the barbecue stand and in the popcorn chicken stand. Analyses of total aldehyde revealed GM concentrations of 163.6, 222.8, and 233.7 μg/m^3^ in western fast food restaurant kitchens, Chinese cafeteria kitchens and street food carts, respectively (Table 1). The aldehyde analyses showed that the three workplaces had similar aldehyde concentration profiles. The most abundant aldehydes were hexaldehyde and nonanal (Supplementary Fig. S1).

**Table 1:** Area concentrations of PAHs and aldehyde in three commercial cooking workplaces.

Species/Chemical	Western fast food kitchen (n = 5)^a^			Chinese cafeteria kitchen (n = 6)			Street food cart (n = 7)		
	Mean(SD)	GM(GSD)	Range	Mean(SD)	GM(GSD)	Range	Mean(SD)	GM(GSD)	Range
**PAH (ng/m** ^**3**^ **)**									
Naphthalene	2890.5 (802.1)	2802.9 (1.3)	(2115.1-3812.2)	3538.9 (1282.8)	3286.0 (1.6)	(1482.8–4757.9)	27102.5 (44973.7)	7541.0 (5.5)	(1154.7–120389.8)
Acenaphthylene	137.4 (147.0)	73.0 (3.8)	(18.2–339.5)	209.8 (86.9)	192.5 (1.6)	(82.9–334.9)	2672.5 (4529.3)	164.5 (28.1)	(<LOD^b^-11256.4)
Acenaphthene	76.1 (49.6)	62.2 (2.1)	(21.4–148.7)	24.3 (25.7)	15.1 (3.2)	(2.2–74.1)	573.5 (806.6)	94.9 (16.1)	(<LOD-2126.5)
Fluorene	87.1 (68.7)	62.7 (2.7)	(15.1–180.1)	95.7 (82.5)	63.1 (3.0)	(11.9–212.1)	1587.6 (2652.4)	64.6 (40.0)	(<LOD-6414.3)
Phenanthrene	70.2 (73.7)	38.1 (3.7)	(7.7–167.9)	79.1 (35.6)	70.5 (1.8)	(27.7–111.8)	1450.5 (2476.2)	36.0 (57.2)	(0.1–5896.3)
Anthracene	8.7 (8.4)	5.3 (3.2)	(1.4–19.9)	11.2 (11.0)	3.4 (12.4)	(0.0–29.0)	256.9 (441.5)	16.6 (20.8)	(<LOD-1097.5)
Fluoranthene	17.8 (11.5)	13.2 (2.7)	(2.9–28.7)	7.8 (5.5)	5.8 (2.8)	(<LOD-14.7)	513.0 (868.4)	16.1 (31.7)	(0.4–1860.8)
Pyrene	22.8 (18.4)	16.0 (2.9)	(3.3–51.1)	12.9 (11.3)	8.3 (3.2)	(1.2–30.5)	499.8 (830.9)	26.3 (24.2)	(0.9–1762.4)
Benzo(a)anthracene	2.9 (2.2)	1.5 (5.4)	(0.1–5.0)	4.2 (3.7)	3.3 (2.4)	(<LOD-8.9)	48.4 (84.1)	9.5 (7.4)	(1.1–223.1)
Chrysene	4.6 (2.6)	4.0 (1.9)	(2.0–7.3)	5.3 (4.7)	3.6 (2.9)	(<LOD-11.1)	76.4 (134.7)	12.9 (8.0)	(1.5–353.5)
Benzo(b)fluoranthene	<LOD			<LOD			34.7 (66.2)	4.4 (9.9)	(<LOD-175.9)
Benzo(k)fluoranthene	<LOD			<LOD			31.8 (57.2)	5.7 (8.7)	(<LOD-155.6)
Benzo(a)pyrene	<LOD			<LOD			27.4 (53.4)	4.0 (8.3)	(<LOD-141.6)
Indeno(1,2,3-cd)pyrene	<LOD			<LOD			6.3 (10.4)	2.6 (4.2)	(<LOD-25.1)
Dibenz(a,h)anthracene	<LOD			<LOD			3.1 (6.2)	1.9 (3.1)	(<LOD-16.4)
Benzo(g,h,i)perylene	<LOD			<LOD			7.7 (14.4)	2.4 (4.9)	(<LOD-37.6)
Total PAH	3318.0 (1133.6)	3171.0 (1.4)	(2371.1–4698.6)	3989.3 (1390.7)	3721.1 (1.6)	(1648.3–5342.0)	34892.2 (56755.4)	8790.2 (6.1)	(1183.5–147585.4)
Total B(a)Peq	3.7 (1.2)	3.6 (1.4)	(2.6–5.4)	4.6 (1.7)	4.2 (1.6)	(1.7–6.2)	80.4 (141.5)	13.5 (8.3)	(1.4–372.3)
**Aldehyde (μg/m** ^**3**^ **)**									
Formaldehyde	15.1 (14.1)	7.6 (4.3)	(0.8–34.7)	12.3 (6.4)	9.2 (3.0)	(1.0–20.2)	35.5 (46.1)	18.0 (3.3)	(7.0–119.2)
Acetaldehyde	18.5 (15.5)	12.1 (3.2)	(1.8–36.2)	42.9 (22.6)	36.3 (2.0)	(9.8–74.5)	17.0 (14.9)	13.8 (1.9)	(8.5–50.0)
Acrolein	6.3 (6.7)	3.2 (3.2)	(<LOD-16.4)	9.2 (10.6)	3.8 (6.1)	(0.2–27.5)	16.4 (15.5)	11.4 (2.4)	(4.7–42.3)
Propinoaldehyde	7.1 (5.7)	5.3 (2.0)	(1.9–16.8)	16.7 (11.3)	13.0 (2.3)	(4.8–29.7)	12.0 (9.1)	10.2 (1.8)	(5.7–32.0)
Crotonaldehyde	12.2 (9.1)	8.6 (2.2)	(2.6–26.6)	3.2 (2.8)	2.6 (2.3)	(<LOD-7.6)	2.7 (2.4)	2.2 (2.3)	(<LOD-6.4)
Butyraldehyde	5.6 (3.8)	3.5 (2.6)	(1.0–10.9)	12.0 (11.8)	7.4 (3.0)	(2.5–28.4)	10.3 (7.2)	8.4 (2.0)	(3.4–24.1)
Valeraldehyde	16.0 (15.2)	5.6 (5.4)	(0.9–35.0)	18.4 (18.6)	10.9 (3.2)	(2.9–44.0)	14.9 (5.8)	13.8 (1.5)	(7.1–23.1)
Hexaldehyde	65.2 (64.7)	27.2 (4.4)	(3.0–164.4)	54.7 (54.4)	34.7 (2.9)	(11.6–142.0)	50.7 (17.6)	48.1 (1.4)	(30.3–69.6)
t-2-Heptenal	33.3 (43.9)	7.9 (6.9)	(<LOD-103.5)	19.2 (25.0)	5.6 (7.4)	(<LOD-59.8)	12.9 (7.6)	10.9 (1.9)	(3.8–24.3)
t,t-2,4-Nonadienal	15.7 (14.9)	6.2 (4.7)	(<LOD-34.7)	8.6 (9.6)	4.8 (3.5)	(<LOD-23.4)	10.5 (10.8)	7.8 (2.1)	(3.5–34.5)
t-2-Nonenal	21.8 (33.1)	4.8 (6.3)	(<LOD-78.2)	9.7 (9.6)	4.9 (4.4)	(0.6–23.8)	3.5 (2.6)	2.7 (2.2)	(1.2–8.0)
t,t-2,4-Decadienal	86.3 (128.0)	15.4 (9.0)	(1.2–307.2)	50.0 (58.6)	17.2 (7.2)	(0.8–143.9)	30.3 (30.6)	19.9 (2.6)	(8.0–75.4)
Nonanal	90.5 (108.7)	24.9 (6.6)	(2.0–265.8)	53.6 (44.3)	40.4 (2.3)	(13.4–131.4)	38.9 (21.0)	34.0 (1.8)	(17.9–73.4)
Total	393.6 (385.7)	163.6 (4.4)	(18.7–946.0)	310.4 (266.5)	222.8 (2.5)	(68.5–725.3)	255.6 (107.0)	233.7 (1.6)	(121.2–407.8)

^a^Sample size.

^b^LOD, limit of detection.

### Personal exposure concentrations

In the western fast food restaurant kitchens and Chinese cafeteria kitchens, half of the participants were female. In the street food carts, however, only one participant was female. No fume extractors were used in street food carts. The prevalence of personal protective equipment and fume extractors differed among the three working environments. The number of working years was shorter in the western fast food restaurant kitchen group compared to the other two groups (Table 2).

**Table 2:** Characteristics of 22 workers from three different workplaces.

Characteristics		Western Fast food kitchen	Chinese cafeteria kitchen	Street food cart
N		12	6	4
		**Mean** ± **SE or N (%)**		
Age (yrs)		22.7 ± 1.3	35.8 ± 4.2	38.3 ± 4.1
Height (cm)		167.9 ± 3.2	164.2 ± 3.9	171.0 ± 1.7
Weight (kg)		60.0 ± 5.6	64.2 ± 5.6	76.7 ± 9.3
Gender	Male	6 (50)	3 (50)	3 (75)
	Female	6 (50)	3 (50)	1 (25)
Education	High school	5 (42)	6 (100)	3 (75)
	College	7 (58)	0	1 (25)
PPE	No	0	2 (33)	2 (50)
	Yes	12 (100)	4 (67)	2 (50)
Fume extractor	No	0	0	4 (100)
	Yes	12 (100)	6 (100)	0
Smoking	No	12 (100)	4 (67)	3 (75)
	Yes	0	2 (33)	1 (25)
Drinking alcohol	No	12 (100)	5 (83)	4 (100)
	Yes	0	1 (17)	0
Working years at this workplace (yrs)		2.3 ± 0.6	11.6 ± 2.3	14.8 ± 6.3

Abbreviation: SE = standard error; yrs = years.

Table 3 shows personal exposure results. In western fast food restaurant kitchens, Chinese cafeteria kitchens and street food carts, the respective GM concentrations of total PAH were 558.5, 4943.1, and 4576.1 ng/m^3^, and the respective GM concentrations of total aldehyde were 67.6, 62.1 and 165.3 μg/m^3^. In terms of PAH ring number wise distribution, the most frequently detected in the three workplaces were two-ring PAHs (78.2–92.3%) followed by three-ring PAHs (6.7–21.3%) and four-ring PAHs (0.4–1.8%) (Supplementary Fig. S2). The three workplaces significantly differed in personal exposure to naphthalene, acenaphthylene, fluorene, anthracene, total PAH, and total BaP_eq_ (p-values 0.006, <0.001, <0.001, 0.020, 0.002, and <0.001 respectively, Supplementary Table S1). In post hoc analysis, the largest differences in PAH concentrations were observed in comparisons between western fast food restaurant kitchens and Chinese cafeteria kitchens and between western fast food restaurant kitchens and street food carts. The aldehyde analyses showed that the three workplace types significantly differed in formaldehyde, acrolein, crotonaldehyde, valeraldehyde, hexaldehyde, t-2-heptenal, t,t-2,4-DDE, nonanal and total aldehyde (p-value range < 0.001–0.018, Supplementary Table S1). Post hoc analysis showed that the largest differences in aldehyde concentrations were observed in comparisons between western fast food restaurant kitchens and street food carts and between Chinese cafeteria kitchens and street food carts. Personal exposure to aldehyde was higher in the street food cart group compared to the other two groups.

**Table 3:** Personal air concentrations of PAHs and aldehydes in three commercial cooking workplaces.

Species/Chemical	Western fast food kitchen (n = 12)			Chinese cafeteria kitchen (n = 6)			Street food cart (n = 4)		
	Mean (SD)	GM (GSD)	Range	Mean (SD)	GM (GSD)	Range	Mean (SD)	GM (GSD)	Range
**PAH (ng/m** ^**3**^ **)**									
Naphthalene^a,b^	1249.0 (1959.9)	338.3 (6.9)	(7.0–6146.7)	4877.2 (3281.0)	3872.6 (2.2)	(1208.8–10009.8)	5707.0 (5306.8)	4297.8 (2.3)	(1922.1–13515.6)
Acenaphthylene^a,c^	21.3 (10.0)	18.7 (1.8)	(3.8–43.8)	537.2 (645.9)	355.8 (2.4)	(186.5–1828.3)	102.1 (94.0)	61.2 (3.6)	(14.0–203.4)
Acenaphthene	20.4 (13.1)	15.1 (2.6)	(2.0–41.5)	262.6 (510.5)	81.7 (11.3)	(<LOD^d^-1283.9)	177.2 (202.8)	24.6 (22.5)	(0.9–372.1)
Fluorene^a,b^	19.9 (16.5)	13.3 (2.7)	(3.4–51.1)	431.3 (665.2)	181.5 (4.1)	(30.5–1758.3)	27.4 (34.0)	229.6 (5.6)	(<LOD-70.1)
Phenanthrene	26.0 (30.1)	19.6 (6.1)	(<LOD^d^-84.4)	83.6 (51.2)	70.9 (1.9)	(29.4–169.4)	134.6 (235.8)	5.8 (52.7)	(0.1–486.5)
Anthracene^b^	3.9 (4.4)	3.4 (10.3)	(<LOD-12.8)	10.4 (4.7)	9.3 (1.8)	(3.2–17.8)	10.9 (16.8)	130.9 (11.3)	(<LOD-35.4)
Fluoranthene	9.5 (8.7)	5.1 (3.9)	(0.5–24.0)	5.6 (3.4)	4.9 (1.7)	(3.0–11.9)	7.6 (11.5)	3.0 (5.1)	(0.5–24.7)
Pyrene	9.2 (8.3)	5.3 (3.5)	(0.7–23.0)	8.1 (3.4)	7.6 (1.5)	(5.1–14.1)	7.5 (12.6)	1.9 (7.1)	(0.3–26.3)
Benzo(a)anthracene	2.5 (2.4)	22.0 (17.8)	(<LOD-7.2)	7.2 (4.4)	5.7 (2.4)	(1.1–14.0)	1.8 (1.8)	0.8 (5.1)	(0.1–3.6)
Chrysene	3.4 (3.8)	46.8 (15.3)	(<LOD-11.9)	10.4 (7.3)	6.3 (4.6)	(0.3–22.4)	7.0 (6.9)	3.7 (4.3)	(0.8–14.1)
Benzo(b)fluoranthene	<LOD			<LOD			<LOD		
Benzo(k)fluoranthene	<LOD			<LOD			<LOD		
Benzo(a)pyrene	<LOD			<LOD			<LOD		
Indeno(1,2,3-cd)pyrene	<LOD			<LOD			<LOD		
Dibenz(a,h)anthracene	<LOD			<LOD			<LOD		
Benzo(g,h,i)perylene	<LOD			<LOD			<LOD		
Total PAH^a,b^	1365.2 (1958.7)	558.5 (4.3)	(58.5–6296.0)	6233.5 (4262.4)	4943.1 (2.2)	(1793.9–12108.4)	6183.1 (5793.9)	4576.1 (2.4)	(1939.4–14638.0)
Total B(a)P_eq_^a,b^	1.7 (2.0)	1.1 (2.4)	(0.4–6.7)	7.1 (3.8)	6.3 (1.7)	(3.4–12.8)	6.5 (6.1)	4.8 (2.4)	(2.0–15.4)
**Aldehyde (μg/m** ^**3**^ **)**									
Formaldehyde^a^	15.1 (6.0)	13.9 (1.6)	(6.4–26.3)	7.8 (6.3)	4.2 (5.0)	(0.2–17.3)	22.0 (3.4)	21.8 (1.2)	(17.1–24.7)
Acetaldehyde	18.8 (6.1)	18.1 (1.3)	(13.0–30.4)	19.4 (11.4)	17.0 (1.7)	(9.4–36.4)	20.8 (4.8)	20.4 (1.3)	(16.1–25.5)
Acrolein^b^	0.5 (0.4)	0.8 (1.5)	(<LOD-1.2)	1.3 (1.3)	1.2 (2.2)	(<LOD-3.0)	3.8 (2.8)	3.2 (1.9)	(1.6–7.9)
Propinoaldehyde	4.9 (1.8)	4.6 (1.5)	(2.2–8.2)	5.3 (3.7)	4.4 (1.9)	(2.5–10.8)	10.9 (6.9)	8.9 (2.2)	(3.0–19.8)
Crotonaldehyde^a,b^	5.9 (1.0)	5.8 (1.2)	(4.6–8.2)	0.9 (1.4)	1.2 (1.8)	(<LOD-3.6)	0.6 (1.1)	1.2 (1.5)	(<LOD-2.2)
Butyraldehyde	2.2 (1.2)	1.9 (1.8)	(0.8–4.5)	2.0 (1.0)	1.8 (1.7)	(0.9–3.2)	6.5 (8.1)	3.4 (4.3)	(<LOD-17.6)
Valeraldehyde^b,c^	0.7 (0.6)	0.9 (1.7)	(<LOD-1.9)	0.7 (0.8)	0.9 (1.6)	(<LOD-2.1)	13.6 (10.8)	9.5 (3.0)	(2.2–26.8)
Hexaldehyde^b,c^	9.9 (3.2)	9.4 (1.4)	(5.9–15.1)	9.1 (3.3)	8.6 (1.5)	(5.2–13.7)	47.5 (33.9)	38.8 (2.1)	(15.0–95.0)
t-2-Heptenal^b,c^	0.4 (0.7)	1.0 (1.4)	(<LOD-1.5)	0.2 (0.5)	1.0 (1.1)	(<LOD-1.3)	7.4 (9.9)	4.2 (3.6)	(<LOD-22.1)
t,t-2,4-Nonadienal	1.2 (1.4)	1.5 (1.7)	(<LOD-3.5)	0.9 (1.5)	1.3 (1.7)	(<LOD-3.5)	3.2 (4.3)	2.4 (3.0)	(<LOD-9.2)
t-2-Nonenal	0.4 (0.6)	0.8 (1.7)	(<LOD-1.6)	1.3 (1.2)	1.2 (2.1)	(<LOD-3.5)	0.6 (1.2)	1.2 (1.5)	(<LOD-2.4)
t,t-2,4-Decadienal^b,c^	2.1 (1.3)	1.9 (1.8)	(<LOD-4.9)	1.1 (1.9)	1.5 (1.9)	(<LOD-4.5)	13.9 (9.7)	11.4 (2.1)	(5.1–26.3)
Nonanal^b^	8.5 (4.4)	7.2 (1.9)	(2.0–16.8)	16.3 (7.9)	14.4 (1.8)	(5.2–28.7)	36.2 (18.7)	32.0 (1.8)	(14.9–52.9)
Total aldehyde^b,c^	70.6 (20.9)	67.6 (1.4)	(40.8–102.3)	66.5 (26.4)	62.1 (1.5)	(37.7–103.4)	187.0 (104.2)	165.3 (1.8)	(83.2–322.2)

^a^Significant difference between western fast food restaurant kitchen and Chinese cafeteria kitchen.

^b^Significant difference between western fast food restaurant kitchen and street food cart.

^c^Significant difference between Chinese cafeteria kitchen and street food cart.

^d^LOD, limit of detection.

### Incremental lifetime cancer risk (ILCR)

**Figure 1:**
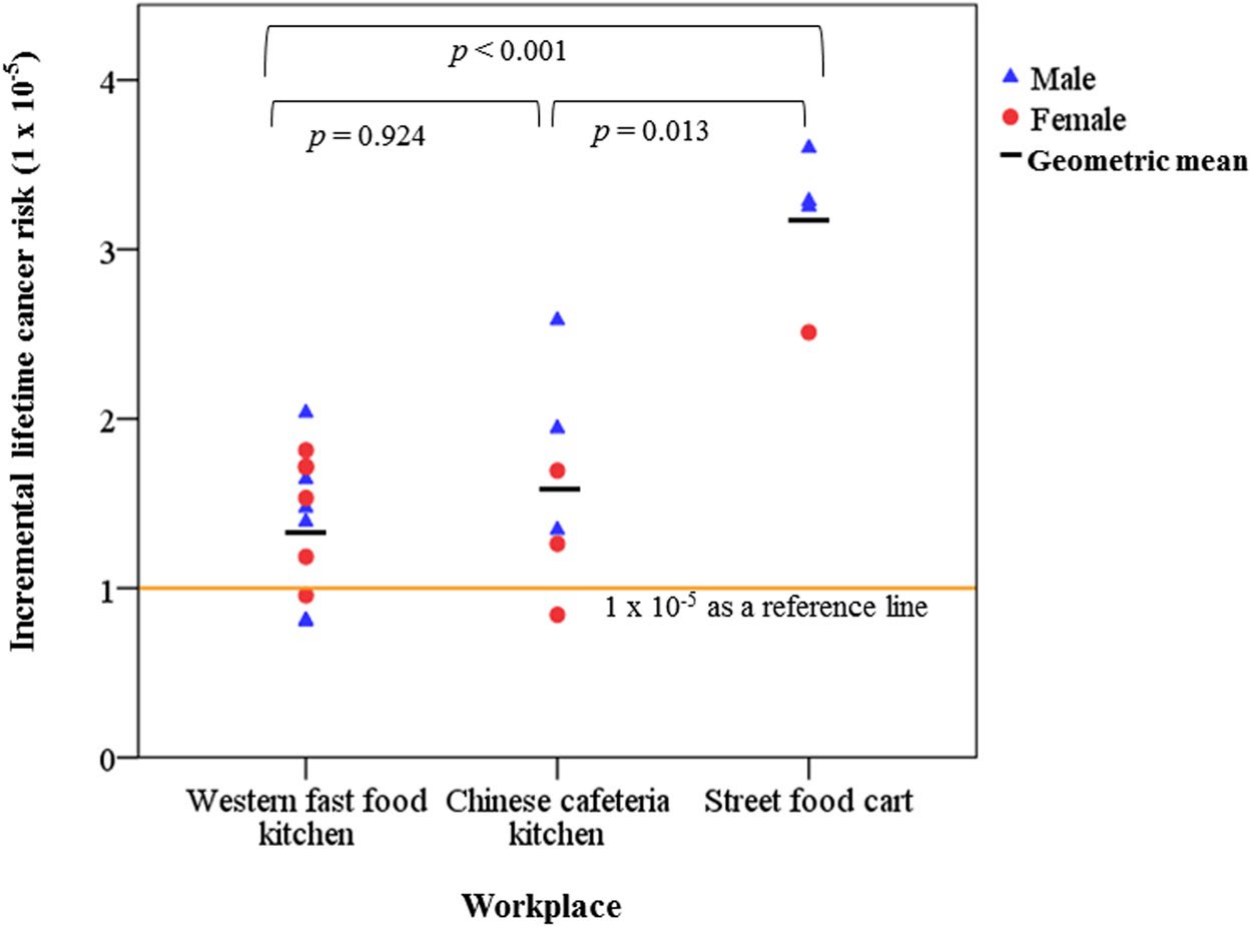
Incremental lifetime cancer risk estimation of workers based on concentrations of PAHs and two aldehydes in three commercial cooking workplaces.

The ILCR ranged from 6.2 × 10^−8^ to 2.7 × 10^−6^ for total PAH and from 6.3 × 10^−6^ to 3.5 × 10^−5^ for aldehydes based on formaldehyde and acetaldehyde (Supplementary Table S2). Thus, total ILCR ranged from 8.0 × 10^−6^ to 3.6 × 10^−5^. The GM (95% confidence interval) ILCR was 1.36 (1.12–1.67) × 10^−5^ in western fast food restaurant kitchens, 1.52 (1.01–2.28) × 10^−5^ in Chinese cafeteria kitchens, and 3.14 (2.45–4.01) × 10^−5^ in street food carts (Fig. 1; Supplementary Table S2). In 18 (82%) workers, the ILCR exceeded 10^−5^. Overall ILCR was significantly higher in street food cart workers compared to western fast food restaurant kitchen workers (*p* < 0.001, power > 0.99) and Chinese cafeteria kitchen workers (*p* = 0.013, power = 0.985) (Fig. 1). However, overall ILCR did not significantly differ between western fast food restaurant kitchen workers and Chinese cafeteria kitchen workers (*p* = 0.924, power = 0.089).

## Discussion

### Exposure concentrations and cooking methods

Workplace selection was based on cooking methods which generate substantial air pollutants, such as frying or barbecuing. Several studies reported these two cooking methods produced more pollutants than the other cooking methods did. For example, Yao, *et al*.^7^ reported that deep frying generates more PAHs compared to pan frying because deep frying uses oil at a hotter temperature and in a larger amount. In Zhao, *et al*.^2^ study, a comparison of night market stalls revealed that total PAH levels were highest in a barbecue food stall (43.145 μg/m^3^). The low indirect heat from burning charcoals used for barbecue cooking can result in incomplete combustion, which is the main mechanism of PAH formation^22^.

The BBQ stand was the only workplace using charcoal as its cooking fuel. Several studies reported that solid fuel use is a significant source of particulate matters and PAHs^10,23,24^. Our findings confirmed the results from the previous studies and showed that the BBQ stand had the highest PAH levels of the investigated workplaces; while its aldehyde level was moderate (Table S3). The highest personal aldehyde levels were found in the popcorn chicken and chicken steak stands which used deep frying as their cooking method. Our findings of aldehyde emissions were similar to those found in Ho *et al*. study^25^, in which a western fast-food chain shop using deep frying as its cooking method had high aldehyde emissions, while a Korean BBQ restaurant had moderate aldehyde emissions. Pollutant emission patterns of barbecuing and deep frying were similar. Both cooking methods generated aldehydes and PAHs with higher aldehyde concentrations in comparison with PAHs. The two chemical species have some compounds with carcinogenic potentials and corresponding cancer risks were estimated using the Eq. 1 accordingly. Both barbecuing and deep frying had similar emission pattern for producing carcinogenic compounds, and are considered as pollutant sources in cooking workplaces.

### Comparison between area and personal concentrations

Correlation coefficients between area and personal GMs for individual workplace measurements were 0.650 (p = 0.058) and 0.383 (p = 0.308) for total PAH and total aldehyde, respectively (Table S3). PAH results showed a moderate correlation with a marginal trend toward significance, while aldehyde results showed no association. A possible explanation for marginal or no associations between area and personal measurements shows as follows. Area measurements were based on samples taken at fixed sites near the stoves whereas personal measurements were based on samples taken from workers who tended to move among various locations throughout the workday. For example, a single worker may have been sampled while in the cooking area, in the preparation area, in the dish-washing station, or in the rest area. Therefore, the carcinogenic risk estimation from personal sampling was more representative compared to those from area samples, which were often used to assess the carcinogenic potencies of cooking emissions in previous studies^10,26,27^.

### Area concentrations

Table 4 shows the PAH concentrations reported in earlier studies. Examples include 1.08–22.8 μg/m^3^ from hot cooking oil fumes^28^; 8.58–89.71 μg/m^3^, 24.7–130 μg/m^3^, and 1.14–7.84 μg/m^3^ in exhaust ducts of restaurants from several Asian studies^10,26,29^; 10–21 μg/m^3^ in commercial kitchens^14^; 1.44–56.9 μg/m^3^ found in five family kitchens^9^, and 24.6–53.4 μg/m^3^ from deep-frying and frying cooking oil fumes under a laboratory fume hood^7^. The PAH levels measured in the current study (1.18–147.59 μg/m^3^) were consistent with those in the literature. The PAH levels in this study were also comparable to levels measured in coke plant workplaces (10.98–146.98 μg/m^3^)^30^, in foundry plant workplaces (77.59 μg/m^3^)^31^, and in smoke from incense burned inside temples (3.35–9.24 μg/m^3^)^32^. Notably, some studies have also reported PAH concentrations in terms of BaP_eq_ concentrations to evaluate their carcinogenic potencies. The range of BaP_eq_ concentrations measured in the current study was 0.001–0.372 μg/m^3^ whereas previous studies have reported ranges of 0.041–0.233 μg/m^3^ for cooking emissions from five family kitchens in Taiwan^9^, 0.023 μg/m^3^ for ion casting emissions in a foundry plant^31^, and 0.20–10.87 μg/m^3^ in emissions from coke plants^30^.

**Table 4:** Comparison of total PAH and BaP_eq_ concentrations (μg/m^3^) in this study with other available data.

Emission source	City	Sampling location/subject	Measured PAHs	Total PAH	BaP_eq_	Reference
Cooking	Kaohsiung, Taiwan	Commercial kitchens, street food carts	16 priority control PAHs: NAP, ACY, ACE, FLU, PHE, ANT, FLUA, PYR, BaA, CHR, BbF, BkF, BaP, IND, DBahA, BghiP	1.18–147.59	0.001–0.372	This study
		Workers of commercial cooking workplaces		0.06–14.6	0.0004–0.0154	This study
		Laboratory	7 PAHs: PHE, FLUA, PYR, BaA, CHR, BbF, BaP	1.08–22.8		Siegmann and Sattler^28^
	Tainan, Taiwan	Commercial kitchens	21 PAHs: 16 priority control PAHs, BeP, PER, COR, CcdP, BbC	24.7–130	0.31–6.7	Li *et al*.^26^
	Hangzhou, China	Commercial kitchens	12 PAHs: NAP, ACE, FLU, PHE, ANT, FLUA, PYR, BaA, CHR, BbF, BkF, BaP, IND, DBahA, BghiP	10–21		Zhu and Wang^14^
	Hong Kong, China	Commercial kitchens	22 PAHs: 16 priority control PAHs, BeP, 9 MA, RET, BaF, PER, COR	1.14–7.84		Chen *et al*.^29^
	Taipei, Taiwan	Workers of food stands at night markets	16 priority control PAHs	23.4–44.2		Zhao *et al*.^2^
	Taiwan	Commercial kitchens	21 PAHs: 16 priority control PAHs, BeP, PER, COR, CcdP, BbC	8.58–89.71	0.06–5.37	Chen *et al*.^10^
	Northern Taiwan	Household kitchens	16 priority control PAHs	1.44–56.9	0.041–0.233	Yu *et al*.^9^
	China	Laboratory	16 priority control PAHs	24.6–53.4		Yao *et al*.^7^
Coking	Kaohsiung, Taiwan	Workers of coke plants	16 priority control PAHs	0.99–6.95		Lin *et al*.^35^
	China	Coke plants	16 priority control PAHs	10.98–146.98	0.20–10.87	Mu *et al*.^30^
Incense burning	Taiwan	Temple	21 PAHs: 16 priority control PAHs, BeP, PER, COR, CcdP, BbC	3.35–9.24		Lin *et al*.^32^
Iron casting	Taiwan	Foundry plant	16 priority control PAHs	77.59 (44.80)	0.023 (0.015)	Chen *et al*.^31^
Traffic	Beijing, China	Traffic police	16 priority control PAHs	5.05	0.082	Liu *et al*.^36^

Abbreviation: 9 MA = 9-methylanthracene; ACE = acenaphthene; ACY = acenaphthylene; ANT = anthracene; BaA = benzo(a)anthracene; BaF = benzo(a)fluoranthene; BaP = benzo(a)pyrene; BbC = benzo(b)chrysene; BbF = benzo(b)fluoranthene; BeP = benzo(e)pyrene; BghiP = benzo(g,h,i)perylene; BkF = benzo(k)fluoranthene; CcdP = cyclopenta[c,d]pyrene; CHR = chrysene; COR = coronene; DBahA = dibenz(a,h)anthracene; FLU = fluorene; FLUA = fluoranthene; IND = indeno(1,2,3-cd)pyrene; NAP = naphthalene; PER = Perylene; PHE = phenanthrene; PYR = pyrene; RET = Retene.

Table 1 shows that the range of total aldehyde concentrations in the three cooking workplaces was 18.7–946.0 μg/m^3^. Concentrations reported in the literature include 159–3095 μg/m^3^ for 13 aldehydes in commercial kitchens^25^, mean concentrations of 185–241 μg/m^3^ for six aldehydes in residential kitchens^33^, and 21–170 μg/m^3^ for 18 carbonyl compounds in five commercial kitchens^27^. Thus, our data were again consistent with the literature. The aldehyde profile in this study showed high concentrations of hexaldehyde, t,t-2,4-DDE and nonanal  detected in COFs, which was consistent with that reported in Peng *et al*.^34^.

### Personal concentrations

Personal concentrations of total PAH measured in the 22 workers in the three commercial cooking workplaces investigated in this study ranged from 0.06 to 14.6 μg/m^3^ (Table 4). Zhao, *et al*.^2^ reported total PAH in a range of 23.4–44.2 μg/m^3^ in cooks at Taiwan night markets (Table 4). As expected, our data for the street food cart workers were consistent with those reported for night market workers due to their similar cooking conditions. In earlier studies of occupational exposure, mean personal concentrations of total PAH were 0.99 and 6.95 μg/m^3^ for side-oven workers and topside-oven workers in a coke plant^35^, respectively, and 5.05 μg/m^3^ for traffic policemen^36^ (Table 4). However, the PAHs measured in our study and for traffic policemen were predominantly 2-ring and 3-ring PAHs whereas PAHs measured in coke oven plants were predominantly 5-ring and 6-ring PAHs, which have a higher toxicity and potency. Mean total BaP_eq_ was also assessed to account for the different toxicities and potencies of PAHs. Mean total BaP_eq_ concentration were 0.004 μg/m^3^ in the current study. In contrast, the BaP_eq_ concentrations reported previously were 0.18 μg/m^3^ for coke side-oven workers, 1.57 μg/m^3^ for coke topside-oven workers, and 0.082 μg/m^3^ for traffic policemen. Therefore, both total PAH and BaP_eq_ concentrations should be investigated and compared to clarify their sources and toxic potencies.

Personal concentrations of the 13 aldehydes measured in this study ranged from 37.7 μg/m^3^ to 322.2 μg/m^3^. In contrast with many studies of personal exposure to PAHs, studies of personal exposure to aldehydes are rare. One example is Svendsen, *et al*.^37^, who reported total personal concentrations of formaldehyde, acetaldehyde and acrolein ranging from 8–186 μg/m^3^ in restaurant kitchens in Norway. Another study of three restaurant kitchens by Sjaastad and Svendsen^11^ reported total personal concentrations of 28.1–154.4 μg/m^3^ for 16 aldehydes and 1.03–17.67 μg/m^3^ for high carbon number aldehydes (C10~C11). The Sjaastad research group also investigated personal aldehyde levels emitted from pan frying (beefsteak) on an electric stove or on a gas stove using various cooking oils in a laboratory kitchen. In studies performed in 2008 and in 2010, the Sjaastad research group reported total aldehyde concentrations in ranges of 129.4–563.4 μg/m^3^ and 81.3–354.4 μg/m^3^, respectively^20,38^. Thus, the exposure levels measured in our study are similar to those reported in the literature.

### Incremental lifetime cancer risk

The ILCR from exposure to cooking emissions ranged from 8.04 × 10^−6^ to 3.60 × 10^−5^ (Table S4). Eighteen (18/22 = 82%) workers in this study had an ILCR higher than 1 × 10^−5^. The percentage contribution to cancer risk was much higher for aldehydes (range, 74.9–99.7%) than for total PAH (range, 0.3–25.1%). The three commercial cooking workplace types had similarity in cooking method (frying) and cooking oil (palm oil and soybean oil). Emissions from western fast food restaurant kitchens and Chinese cafeteria kitchens were regulated by Taiwan’s Air Pollution Control Act^39^. Under the Act, these kitchens needed to install air pollution control devices, maintain the devices under workable conditions, clean the devices regularly, and meet the emission standards. Street food carts had an exception from the Taiwan’s Air Pollution Control Act due to their small scale (capital investment < NT$ 100,000, and workplace area < 100 m^2^). This lead to air pollutants from street food carts uncontrolled. This could be the reason that workers in the street food cart group had significantly higher overall ILCRs compared to workers in the other two groups. One of the notable differences of these three types was exhaust systems. There was no effective mechanical exhaust systems in the street food cart group. Other studies have also indicated the need for effective ventilation systems to control cooking-related emissions. For example, a comparison of kitchens by Svendsen, *et al*.^37^ found that concentrations of fat aerosols and aldehyde concentrations were highest in kitchens with insufficient exhaust systems; another study by Yu, *et al*.^9^ similarly reported high PAH concentrations in kitchens with poor ventilation. Although the open air environment of street food carts enables rapid dispersal of pollutants, cooking emissions were higher in the street food cart group compared to the other two groups probably because street food carts are rarely equipped with fume extractors or other exhaust systems for collecting and removing pollutants from breathing zones. Additionally, other factors, such as equipment type, maintenance, cooking space etc. may be potential contributors for high PAH and aldehyde concentrations of street food cart workers.

In the literature, reported ranges of estimated cancer risk related to cooking emissions include 2.5 × 10^−6^ – 1.4 × 10^−5^ for household cooking emissions^9^, 9.0 × 10^−5^– 1.13 × 10^−4^ for night market workers^2^, and 1.96 × 10^−8^–1.31 × 10^−6^ for frequent customers of six restaurants^27^. For comparison, examples of median or mean ILCRs reported in the literature included 2.96 × 10^−5^ for inhalation exposure in temple workers^40^; 1.055 × 10^−4^ for traffic assistants (i.e., workers who assist police in controlling heavy traffic)^41^, 1.6 × 10^−5^ in motorcycle commuters based on an estimated daily BaP_eq_ of 0.40 μg/d^23^, and 2.3 × 10^−4^ in topside coke oven workers based on personal exposure to 16 PAHs^35^. In comparison with other sources, cooking-related emissions showed a moderate cancer risk.

Aldehyde species are dominant chemical species in cooking-related emissions. The low carbon number aldehydes formaldehyde and acetaldehyde are confirmed and probable carcinogens, respectively^6^. Some high carbon number aldehydes also showed carcinogenic effects. For example, Wu, *et al*.^21^ reported that some high carbon number aldehydes (e.g., t,t-2,4-DDE, t,t-2,4-nonadienal, t-2-decenal and t-2-undecenal) were potent mutagens. The aldehyde t,t-2,4-DDE promotes cancer by inducing cancer cell proliferation and by inhibiting anti-oxidant enzyme activities^19,42–44^. However, data are insufficient for their classification as probable or possible carcinogens. High carbon number aldehydes were not be able to include in the risk analyses in the current study. Notably, the cancer risk might be even higher than the estimated values.

### Limitations and strengths

This study has several limitations. First, since the street food carts were located on the roadside, vehicular emissions may have resulted in overestimated cooking emissions. On the other hand, the street food carts analyzed in this study were located on a street with a low traffic volume of ~5600 vehicles per day (Directorate General of Highways, 2014)^45^. The estimated total PAH emission of this traffic volume was 0.232 μg/m^3^ based on Ho, *et al*.^46^ findings which indicated total PAH emission of 2.211 μg/m^3^ at the traffic volume of 53000 vehicles per day. Therefore, the influence of automobile exhaust on these workplaces was probably limited. Nevertheless, future studies should recognize and control for the effects of traffic emissions when investigating cooking workplaces located on or near the roadside. Second, the findings of this study cannot be generalized to all commercial cooking workplaces because the analysis was limited to small or medium sized workplaces in which the primary cooking method was frying or barbecuing. For a comprehensive study of the PAH and aldehyde levels in cooking workplaces and for estimates of cancer risks in populations with high exposure to cooking-related emissions, future studies should include analysis of large-sized cooking workplaces, which may differ in terms of number and type of cooking activities, and in terms of the number of workers. Third, the small sample size precluded assessment of other contributing factors in exposure to PAH and aldehyde (e.g., cigarette smoking and cooking oil type). The small sample size also precluded assessment of gender-specific exposure and gender-specific cancer risk in the three groups. Further gender-specific analyses are needed because COF exposure associated with lung cancer in nonsmoking women. Despite the small sample size, however, this study demonstrated a significantly higher cancer risk in street food cart workers compared to workers in the other two groups.

This study is the first to estimate the cancer risk of personal exposure to both PAHs and aldehydes in cooking workplaces. The estimation was more representative of human exposure compared to previous studies that have used area samples to assess the carcinogenic potencies of cooking emissions^10,26,27^. This study investigated occupational exposure to cooking-related emissions and revealed that workers in the street food cart group had the highest ILCRs. Notably, aldehyde species were the main contributors to cancer risk. Although the street food cart group belongs to the small-scaled workplaces, it accounts for one third of all cooking workplaces, and the cooking emissions from street food carts affect not only workers, but also people in nearby areas. The considerable emissions generated from street food carts is problematic not only in Taiwan, but in many countries throughout the world. The data in this study suggest that cooking emissions and exposure from street food carts can be effectively controlled by installing mechanical exhaust systems and maintaining them in good condition.

## Methods

### Workplace selection and sampling strategy

The three commercial cooking workplace types analyzed in this study were kitchens in western fast food restaurant chains (main product: fried chicken), kitchens in cafeteria-style Chinese restaurant chains, and street food carts. These workplace types were selected for analysis because the cooking methods that they use (frying or barbecuing) are known to generate substantial air pollutants^23,34^. Additionally, western fast food restaurant kitchens, cafeteria-style Chinese restaurant kitchens, and street food carts account for most of the commercial cooking workplaces in Taiwan (10%, 21%, and 32% of respectively)^47^. These cooking workplaces are associated with high personal exposure to cooking-related emissions. Table 5 shows the characteristics and sample sizes of the three cooking workplace types. The analysis included three restaurants or vendors in each of the three groups. All western fast food restaurant kitchens and Chinese cafeteria kitchens analyzed in this study followed Taiwan’s Air Pollution Control Act^39^; which regulated COFs of commercial kitchens by installing exhaust ventilation systems (hoods, ducts, air cleaning devices, and fans) above the frying units or stoves (Supplementary Fig. S3), maintaining the exhaust systems under workable conditions, and cleaning the system regularly. Street food carts were not obligated to install air pollution control devices due to their small scale in terms of capital investment and working counter area (1.5–3.0 m^2^), while some used wall-mounted fans to increase general ventilation (Supplementary Fig. S4). The room inside the cart was used for storage and to house the cooking machinery. Workers stood outside of the carts to prepare food.

**Table 5:** Characteristic information of three commercial cooking workplaces.

Workplace/sampling conditions	Western fast food kitchen (n = 3)			Chinese cafeteria kitchen (n = 3)			Street food cart^a^ (n = 3)		
	FK1	FK2	FK3	CK1	CK2	CK3	FS1	FS2	FS3
Area (m^2^)^b^	20.0	20.0	21.6	51.1	40.8	35.4	3.0	3.0	1.5
Main cooking method	Pan-frying			Stir-frying			Deep-frying	Deep-frying	Barbecuing
	Deep-frying			Pan-frying					
				Deep-frying					
Type of cooking oil	palm oil			palm oil			soy bean oil	soy bean oil	
				soy bean oil					
Oil consumption (L/d) (Mean ± SD)	22.9 ± 3.2			14.3 ± 2.1			5.7 ± 1.2	11.4 ± 1.8	NA^d^
Cooking fuel	LPG^c^			LPG			LPG	LPG	Charcoal
Exhaust hood length (cm)	280	100	220	320	300	280	NA	NA	NA
Exhaust hood width (cm)	120	100	110	110	100	80	NA	NA	NA
Exhaust flow rate (m^3^/min)	20	26	26	29	32	27	NA	NA	NA
Sample size									
area sampling	1	2	2	2	2	2	3	2	2
personal sampling	4	4	4	2	2	2	2	1	1

^a^These included carts for providing Taiwanese popcorn chicken, fried chicken fillet, and barbecued meat.

^b^For fast food and Chinese cafeteria kitchens, it means workplace area. As for the street food cart group, it is area of food counter.

^c^Liquefied petroleum gas.

^d^No available information.

Between September 4, 2014 and November 1, 2014, area and personal samples were collected to determine concentrations of PAHs and aldehydes (Supplementary Fig. S5). For each restaurant or vendor, all samples were collected for 8 h on the same day. Area samples were collected in the kitchen area or other working area at a height of 1.5 m to represent the breathing zone of a worker in a standing position. One to three area samples were taken at each restaurant or vendor; thus, five, six and seven samples were taken in western fast food restaurant kitchens, Chinese cafeteria kitchens, and street food carts. Personal samples were taken from workers who were responsible for cooking. The recruited workers included twelve western fast food restaurant kitchen workers, six Chinese cafeteria kitchen workers, and four street food cart workers (Table 5). All subjects gave written informed consent to participate before the study was performed. Each participating worker wore two personal samplers (one for PAHs and one for aldehydes) and completed questionnaires regarding personal demographic data and characteristics pertinent to this study, including lifestyle (e.g., tobacco and alcohol consumption), occupational history (e.g., work history, periods of employment, cooking method used, and protective equipment used), and health status (e.g., chronic disease, respiratory disease). The study protocol (IRB number: KMUH-IRB-990191) received ethical approval by the Institutional Review Board of Kaohsiung Medical University Hospital (IRB-KMUH). The study was performed in accordance with the guidelines and regulations of the IRB-KMUH. Names and other personal information that could be used to identify participants were excluded from the manuscript, including Supplementary Information.

### Sampling and analysis methods

This study targeted the 16 PAHs prioritized for control by the United States Environmental Protection Agency (Supplementary Table S5) due to their high toxicity and high potential for human exposure. Particulate matter was collected with a Teflon filter (25 mm × 2 μm pore size, Pall Corporation, Port Washington, New York, USA) in an IOM (Institute of Occupational Medicine, Edinburgh, U.K.) sampler, and gaseous PAHs were collected with a polyurethane foam cartridge (ORBO™ 1000, 22 mm (OD) × 7.6 cm (L), Supelco, Bellefonte, Pennsylvania, USA). Particulate- and gas-phase PAHs were simultaneously collected with a specially designed single-pump sampling train. The IOM sampler had a Teflon filter in the front section of the sampling train. One tube was connected to a polyurethane foam cartridge, and another tube was connected to a flow regulator. The two tubes converged at a tee fitting connected to a pump^34^ (Supplementary Fig. S6). The flow regulator was adjusted to obtain the flow rates required by the particulate- and gas-phase sampling devices (2 L/min and 1 L/min, respectively). The flowrates were measured and confirmed by an air flow calibrator (Defender 510, Mesa Laboratories, Inc., Butler, NJ, USA). Collected samples were extracted using a 1:1 mixture of acetone and hexane in a microwave extraction system (START E, Milestone, Shelton, CT, USA)^24,48^. After extraction, the samples were analyzed by a gas chromatograph/mass spectrometer (Trace GC Ultra/DSQ II MS, Thermo Fisher Scientific, Waltham, Massachusetts, USA). The PAHs were separated with a capillary gas chromatography column (Equity®-5, 30 m × 0.25 mm × 0.25 μm, Supelco, Bellefonte, Pennsylvania, USA) with a temperature program. The analytical quality controls for the 16 PAHs are shown in Supplementary Table S5.

The thirteen aldehydes selected for analysis in this study (Supplementary Table S6) were targeted because they have raised health concerns (e.g., formaldehyde and acetaldehyde) or because their chemical characteristics are representative of COFs^34^ (e.g., nonanal and t,t-2,4-DDE). The sampling train used to collect aldehydes was identical to that used to collect PAHs, i.e., an IOM sampler with a DNPH-coated glass fiber filter (25 mm × 2 μm pore size, Supelco, Bellefonte, Pennsylvania, USA) and a 2,4-DNPH cartridge (Supelco, Bellefonte, Pennsylvania, USA) were connected in series for particulate- and gaseous- phase aldehyde sampling. After sample collection, the samplers were stored at 4 °C in sealed aluminum bags until extraction. Samples were extracted by acetonitrile and then analyzed with a high performance liquid chromatograph (PU-2089, Jasco, Japan) equipped with an ultraviolet detector operated at 360 nm (Varian ProStar 320, Varian, USA). A reverse phase column (Ascentis® RP-Amide Column, 5 μm, 250 × 4.6 mm, Supelco, Bellefonte, Pennsylvania, USA) with a gradient mobile phase of acetonitrile/water was used to separate aldehydes. The gradient program was performed at a flowrate of 1.2 ml/min as follows: start with an acetonitrile/water ratio of 40/60 for 1 min, increase acetonitrile from 40% to 90% within 49 min, hold for 3 min, and decrease acetonitrile from 90% to 40% within 12 min. The analytical quality controls for the 13 aldehydes are shown in Supplementary Table S6.

### Estimation of ILCR

For each participant, ILCR was estimated according to the risk assessment guidelines established by US Environmental Protection Agency^49^, which are used extensively in the literature, including our previous works^9,27,50^. The equation used to calculate ILCR was

1 $${\rm{ILCR}}=({\rm{C}}\cdot {\rm{IR}}\cdot {\rm{ET}}\cdot {\rm{ED}}\cdot {\rm{SF}})/({\rm{BW}}\cdot {\rm{LT}})$$

where C is the concentration (μg/m^3^) of a pollutant in air, IR is the inhalation rate (0.83 m^3^/h for males and 0.69 m^3^/h for females), ET is exposure time (8 h, i.e., work hours per day), ED is exposure duration (40 years, i.e., age 20 to 60 years), BW is body weight (69.2 kg for males and 55.5 kg for females)^9^, LT is lifetime (70 years), and SF is slop factor ([μg/kg/day]^−1^). The slop factors for PAH, formaldehyde and acetaldehyde are 0.0039, 0.000021, and 0.00001, respectively^51^.

The ILCR was calculated for all 16 PAHs and for two aldehyde chemicals (formaldehyde and acetaldehyde). For each worker, total ILCR was obtained by adding the 18 ILCR values.

### Statistical Analysis

The PAH concentrations, aldehyde concentrations, and total ILCR data were log-normal distributed; therefore, they were log-transformed. The ANOVA test was then used to analyze the concentration or risk difference in the three commercial cooking types, and the Bonferroni method was used for post-hoc analysis. These statistical analyses were performed with IBM SPSS Statistics 21 software (IBM SPSS Inc., Armonk, NY, USA). All *p* values were two-tailed, and *p* values < 0.05 were considered statistically significant. Statistical power calculation was performed with R (R version 3.4.3).

## Supplementary information

Supplementary information
