# Supplementary material for: Risk assessment of personal exposure to polycyclic aromatic hydrocarbons and aldehydes in three commercial cooking workplaces
Source: Sci Rep. 2019 Feb 7;9:1661. doi: 10.1038/s41598-018-38082-5 (PMC6367358; doi:10.1038/s41598-018-38082-5)
Supplement: Supplementary file 1 — Supplementary information [file 41598_2018_38082_MOESM1_ESM.pdf]

Risk assessment of personal exposure to polycyclic aromatic hydrocarbons and aldehydes in three commercial cooking workplaces

Ming-Tsang Wu, Pei-Chen Lin, Chih-Hong Pan, Chiung-Yu Peng*

Supplementary information

Figures

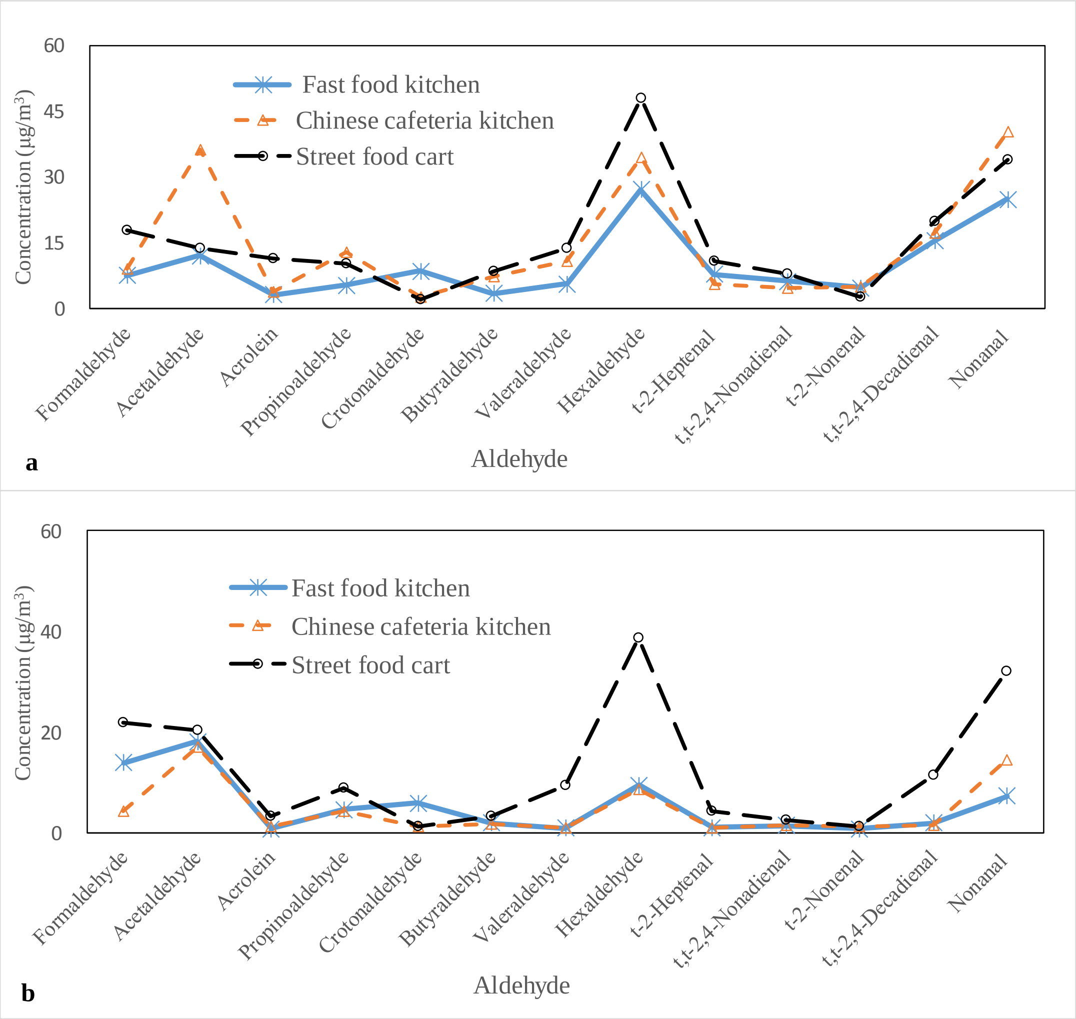

Figure S1. Geometric mean levels of aldehydes for (a) area measurements and (b) personal measurements.

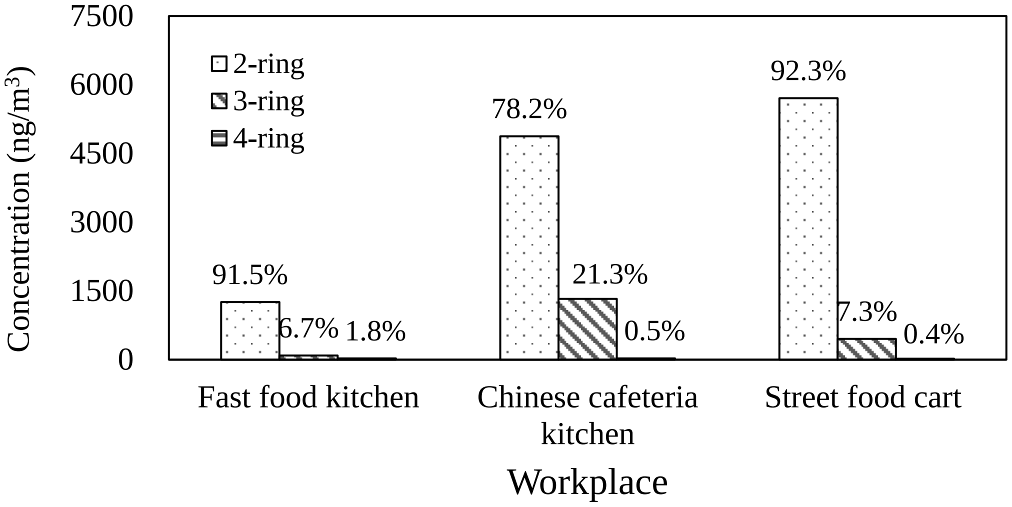

Figure S2. Ring number wise distribution of personal air concentrations of PAHs in three commercial cooking workplaces.

2-ring: naphthalene; 3-ring: acenaphthylene, acenaphthene, fluorene, phenanthrene, anthracene; 4-ring: fluoranthene, pyrene, benzo(a)anthracene, chrysene

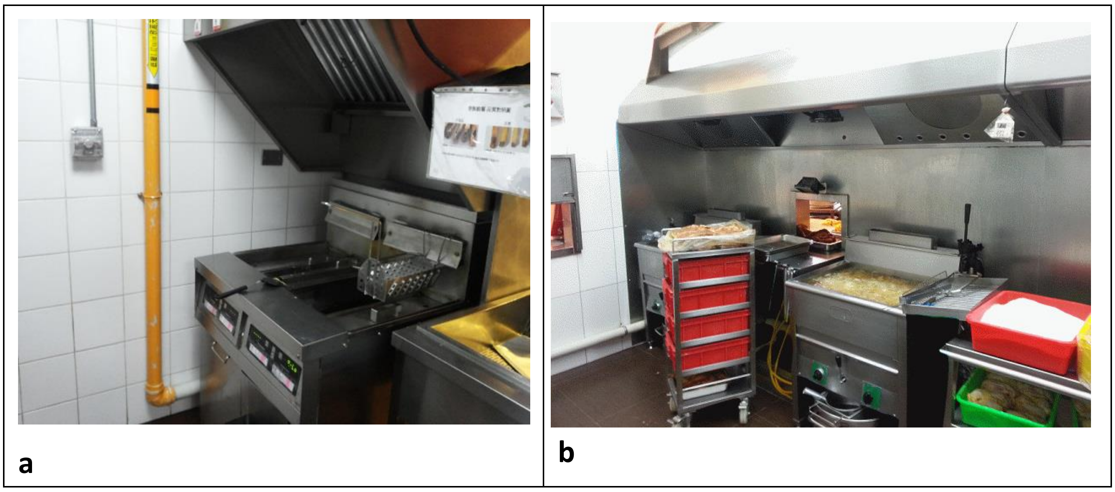

Figure S3. Exhaust ventilation system in the Western fast food kitchen (a) and the Chinese cafeteria kitchen (b).

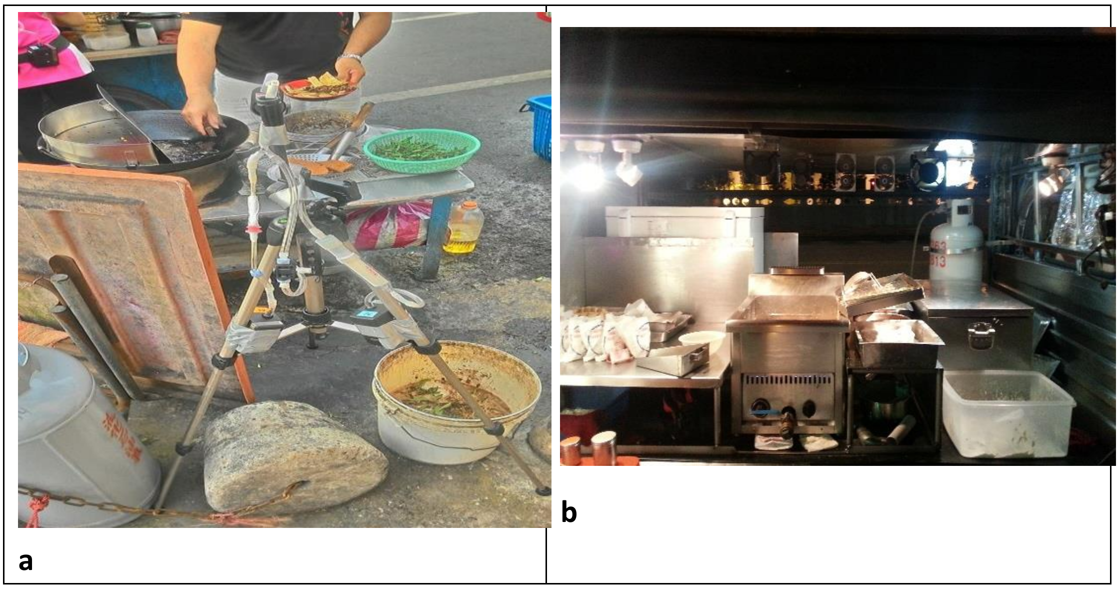

Figure S4. Ventilation status in the Taiwanese popcorn chicken cart (a), and the fried chicken fillet cart (b).

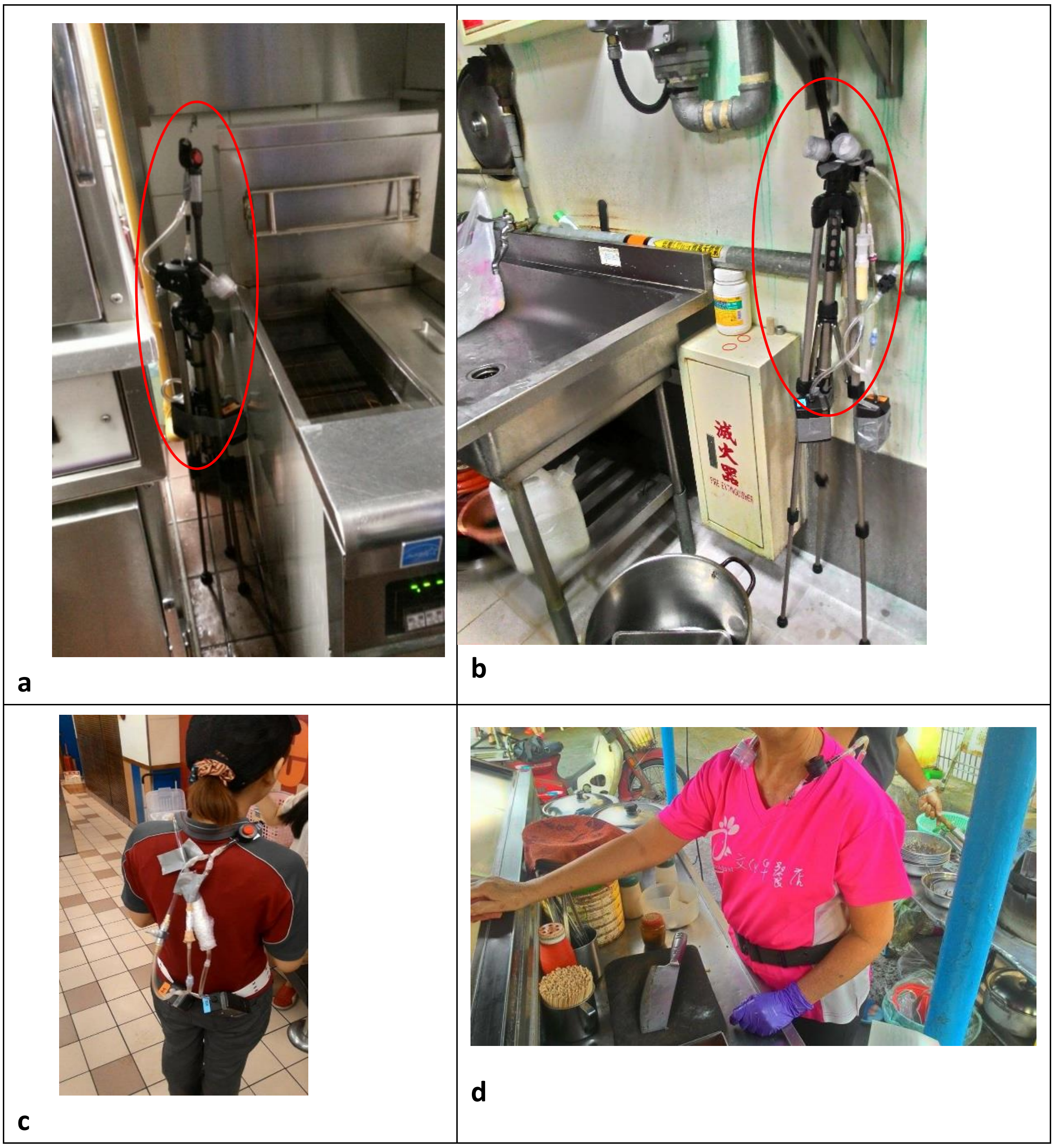

Figure S5. Kitchen area sampling at the western fast food kitchen (a), kitchen area sampling at the Chinese cafeteria kitchen (b), personal air sampling at the fast food kitchen (c), and personal sampling at a street food cart (d).

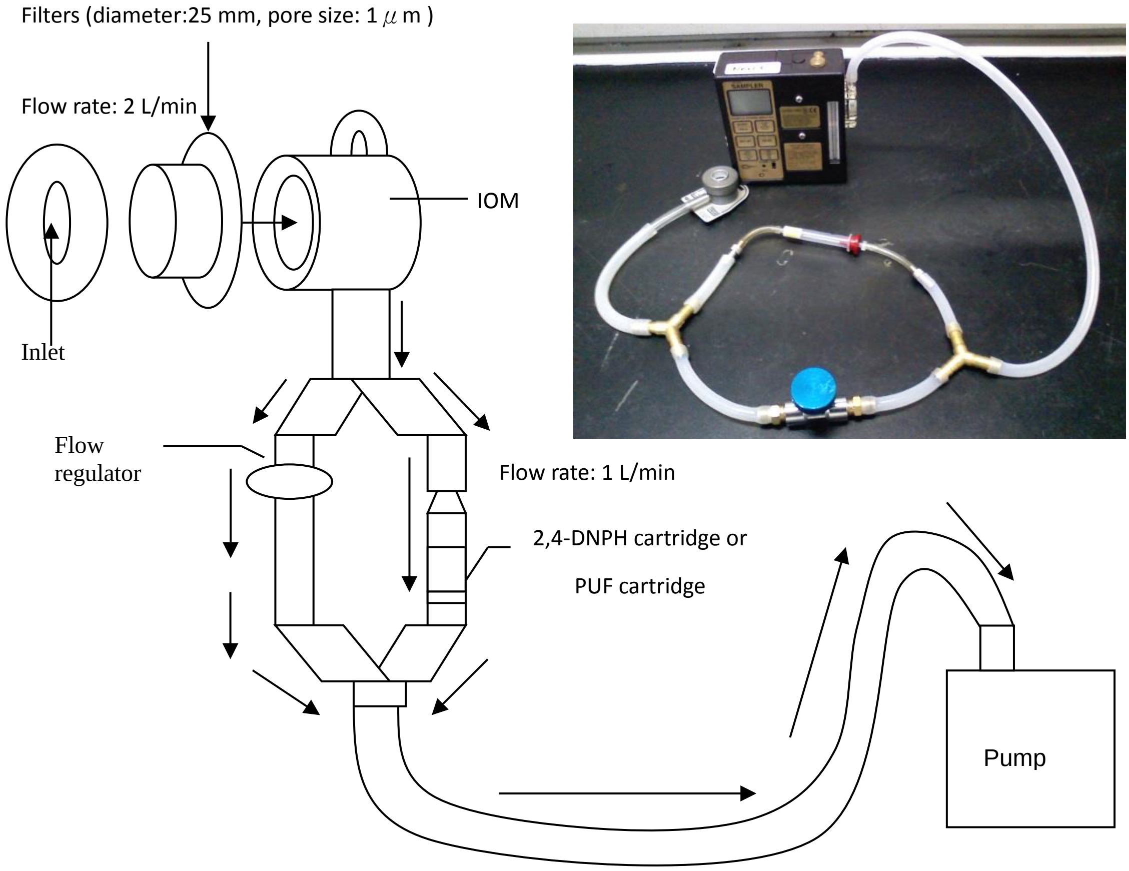

Figure S6. A Schematic plot of the sampling train (Peng et al., 2017).

Tables

Table S1. Comparison results in terms of *p* values for personal air concentrations of PAHs and aldehydes among three commercial cooking workplaces

Species / Chemical	Three groups	Fast food kitchen vs Chinese cafeteria kitchen	Fast food kitchen vs street food cart	Chinese cafeteria kitchen vs street food cart
PAH				
Naphthalene	**0.006**	**0.015**	**0.028**	0.994
Acenaphthylene	**p <0.001**	**p <0.001**	0.056	**0.010**
Acenaphthene	0.229	0.202	0.896	0.598
Fluorene	**p <0.001**	**0.001**	**0.002**	0.955
Phenanthrene	0.207	0.460	0.593	0.189
Anthracene	**0.020**	0.592	**0.015**	0.137
Fluoranthene	0.748	0.998	0.737	0.809
Pyrene	0.252	0.832	0.368	0.235
Benzo(a)anthracene	0.068	0.488	0.061	0.421
Chrysene	0.098	0.212	0.160	0.932
Benzo(b)fluoranthene	NA[a]			
Benzo(k)fluoranthene	NA			
Benzo(a)pyrene	NA			
Indeno(1,2,3-cd)pyrene	NA			
Dibenz(a,h)anthracene	NA			
Benzo(g,h,i)perylene	NA			
Total PAH	**0.002**	**0.006**	**0.020**	0.995
Total $B(a)P_{eq}$	**p <0.001**	**0.001**	**0.014**	0.860
Aldehyde				
Formaldehyde	**0.018**	**0.039**	0.665	**0.027**
Acetaldehyde	0.759	0.947	0.844	0.741
Acrolein	**0.002**	0.305	**0.001**	**0.039**
Propinoaldehyde	0.097	0.981	0.108	0.126
Crotonaldehyde	**p <0.001**	**p <0.001**	**p <0.001**	0.998
Butyraldehyde	0.409	0.980	0.444	0.434
Valeraldehyde	**p <0.001**	0.986	**p <0.001**	**p <0.001**
Hexaldehyde	**p <0.001**	0.924	**p <0.001**	**p <0.001**
t-2-Heptenal	**0.001**	0.999	**0.001**	**0.004**
t,t-2,4-Nonadienal	0.332	0.964	0.377	0.349
t-2-Nonenal	0.237	0.317	0.396	0.999
t,t-2,4-Decadienal	**p <0.001**	0.678	**p <0.001**	**p <0.001**
Nonanal	**0.002**	0.098	**0.002**	0.152
Total aldehyde	**0.002**	0.900	**0.002**	**0.003**

[a] NA, no available information

Table S2. Cancer risk ($x10^{-5}$) estimation of personal PAH and aldehyde intake in three types of commercial cooking workplaces.

Species / Chemical	Western fast food kitchen (n=12)			Chinese cafeteria kitchen (n=6)			Street food cart (n=4)		
	Mean (SD)	GM (GSD)	Range	Mean (SD)	GM (GSD)	Range	Mean (SD)	GM (GSD)	Range
PAH									
Naphthalene	0.0160 (0.026)	0.0044 (6.75)	(0.0001- 0.0809)	0.0802 (0.0547)	0.0632 (2.24)	(0.0195- 0.1658)	0.1009 (0.0934)	0.0763 (2.29)	(0.0349- 0.2385)
Acenaphthylene	0.0003 (0.000)	0.0002 (1.88)	(0.0000- 0.0006)	0.0089 (0.0107)	0.0058 (2.46)	(0.0030- 0.0303)	0.0018 (0.0017)	0.0011 (3.61)	(0.0003- 0.0036)
Acenaphthene	0.0003 (0.000)	0.0002 (2.61)	(0.0000- 0.0006)	0.0043 (0.0085)	0.0026 (36.22)	(<LOD- 0.0213)	0.0031 (0.0036)	0.0004 (22.22)	(0.0000- 0.0066)
Fluorene	0.0003 (0.000)	0.0002 (2.89)	(0.0000- 0.0008)	0.0071 (0.0110)	0.0030 (4.18)	(0.0005- 0.0291)	0.0005 (0.0006)	0.0305 (56.64)	(<LOD- 0.0012)
Phenanthrene	0.0004 (0.000)	0.0004 (17.17)	(<LOD[c]- 0.0013)	0.0014 (0.0008)	0.0012 (1.90)	(0.0005- 0.0027)	0.0024 (0.0042)	0.0001 (52.16)	(0.0000- 0.0086)
Anthracene	0.0005 (0.001)	0.0005 (16.91)	(<LOD- 0.0016)	0.0017 (0.0008)	0.0015 (1.75)	(0.0005- 0.0029)	0.0019 (0.0030)	0.0550 (30.01)	(<LOD- 0.0063)
Fluoranthene	0.0001 (0.000)	0.0001 (3.91)	(0.0000- 0.0003)	0.0001 (0.0001)	0.0001 (1.71)	(0.0000- 0.0002)	0.0001 (0.0002)	0.0001 (5.01)	(0.0000- 0.0004)
Pyrene	0.0001 (0.000)	0.0001 (3.53)	(0.0000- 0.0003)	0.0001 (0.0001)	0.0001 (1.47)	(0.0001- 0.0002)	0.0001 (0.0002)	0.0000 (7.04)	(0.0000- 0.0005)
Benzo(a)anthracene	0.0032 (0.003)	0.0257 (15.87)	(<LOD- 0.0092)	0.0116 (0.0070)	0.0093 (2.35)	(0.0019- 0.0226)	0.0031 (0.0031)	0.0015 (5.04)	(0.0002- 0.0063)
Chrysene	0.0004 (0.000)	0.0140 (44.19)	(<LOD- 0.0015)	0.0017 (0.0012)	0.0010 (4.55)	(0.0001- 0.0036)	0.0012 (0.0012)	0.0007 (4.25)	(0.0001- 0.0025)
Benzo(b)fluoranthene	NA[c]			NA			NA		
Benzo(k)fluoranthene	NA			NA			NA		
Benzo(a)pyrene	NA			NA			NA		
Indeno(1,2,3-cd)pyrene	NA			NA			NA		
Dibenz(a,h)anthracene	NA			NA			NA		
Benzo(g,h,i)perylene	NA			NA			NA		
Sub-total	0.022 (0.026)	0.014 (2.36)	(0.0062- 0.0880)	0.1171 (0.0645)	0.103 (1.76)	(0.0550- 0.2114)	0.1153 (0.1076)	0.085 (2.42)	(0.0356- 0.2714)
Aldehyde									
Formaldehyde	0.88 (0.35)	0.81 (1.56)	(0.37- 1.51)	0.68 (0.54)	0.37 (4.92)	(0.02- 1.50)	2.10 (0.30)	2.08 (1.17)	(1.67- 2.34)
Acetaldehyde	0.52 (0.17)	0.50 (1.35)	(0.37- 0.83)	0.81 (0.47)	0.71 (1.74)	(0.39- 1.50)	0.95 (0.21)	0.93 (1.26)	(0.73- 1.16)
Sub-total	1.40 (0.40)	1.34 (1.37)	(0.80- 2.01)	1.49 (0.64)	1.37 (1.60)	(0.63- 2.50)	3.05 (0.43)	3.02 (1.16)	(2.47- 3.50)
Total[a,b]	1.42 (0.40)	1.36 (1.37)	(0.80- 2.03)	1.61 (0.61)	1.52 (1.47)	(0.84- 2.58)	3.16 (0.46)	3.14 (1.17)	(2.51- 3.60)
[95% CI]	[1.17-1.68]	[1.12-1.67]		[0.97-2.25]	[1.01-2.28]		[2.43-3.90]	[2.45-4.01]	

[a] Significant difference between fast food kitchen vs Chinese cafeteria kitchen
[b] Significant difference between Chinese cafeteria kitchen vs street food cart
[c] NA, No available information

Table S3. Arithmetic means, geometric means and correlation of area and personal measurements for total PAH and total aldehyde in cooking workplaces

Item	Arithmetic mean		Geometric mean	
	Total PAH (μg/m^3)	Total aldehyde (μg/m^3)	Total PAH (μg/m^3)	Total aldehyde (μg/m^3)
**Area**				
Fast food kitchen-1	2.46	34.94	2.46	30.94
Fast food kitchen-2	2.52	558.37	2.51	401.79
Fast food kitchen-3	4.55	416.18	4.54	351.97
Chinese cafeteria kitchen-1	2.34	431.91	2.23	317.32
Chinese cafeteria kitchen-2	5.13	159.75	5.13	131.22
Chinese cafeteria kitchen-3	4.23	339.65	4.50	265.46
Popcorn chicken	2.43	162.30	2.22	153.34
Chicken steak stand	6.06	287.40	5.71	284.45
BBQ stand	112.41	363.91	106.78	360.99
**Personal**				
Fast food kitchen-1	0.12	46.90	0.12	46.69
Fast food kitchen-2	3.38	78.50	2.72	77.36
Fast food kitchen-3	0.59	86.41	0.55	85.70
Chinese cafeteria kitchen-1	1.79	80.12	1.79	80.17
Chinese cafeteria kitchen-2	4.25	44.12	3.77	43.65
Chinese cafeteria kitchen-3	9.03	76.82	8.30	72.06
Popcorn chicken	2.47	227.39	2.41	206.54
Chicken steak stand	5.16	210.15	5.16	210.38
BBQ stand	14.64	83.24	14.62	83.18
Correlation coefficient	0.667	0.233	0.650	0.383
p-value	0.050	0.546	0.058	0.308

Table S4. Cancer risk estimation for individual worker

			Cancer risk (x 10^{-5})															% contribution	
ID	Group[a]	Gender[b]	NAP	ACY	ACE	FLU	PHE	ANT	FLUA	PYR	BaA	CHR	TPAH	FA	AA	Tald	Total	Ald (%)	PAH (%)
1	1	1	0.0076	0.0002	0.0002	0.0002	0.0005	0.0008	0.0002	0.0002	NA[c]	NA	0.0098	0.8662	0.5152	1.3813	1.3911	99.3%	0.7%
2	1	1	0.0050	0.0004	0.0005	0.0006	0.0008	0.0016	0.0003	0.0003	NA	NA	0.0095	0.8020	0.8313	1.6333	1.6428	99.4%	0.6%
3	1	1	0.0152	0.0002	0.0003	0.0001	0.0001	0.0000	0.0000	0.0000	0.0008	0.0003	0.0170	1.0831	0.3748	1.4580	1.4749	98.9%	1.1%
4	1	1	0.0161	0.0002	0.0002	0.0001	0.0001	0.0001	0.0001	0.0001	0.0039	0.0006	0.0215	1.5117	0.5015	2.0131	2.0346	98.9%	1.1%
5	1	1	0.0001	0.0002	0.0001	0.0001	0.0000	0.0003	0.0000	0.0000	0.0092	0.0015	0.0115	0.3655	0.4336	0.7991	0.8106	98.6%	1.4%
6	1	1	0.0011	0.0000	0.0001	0.0001	0.0000	0.0002	0.0000	0.0000	0.0041	0.0005	0.0062	0.4007	0.3975	0.7982	0.8044	99.2%	0.8%
7	1	2	0.0568	0.0002	0.0003	0.0000	0.0001	0.0000	0.0001	0.0001	0.0054	NA	0.0630	1.2657	0.3856	1.6512	1.7142	96.3%	3.7%
8	1	2	0.0051	0.0004	0.0006	0.0008	0.0010	0.0016	0.0003	0.0002	NA	NA	0.0101	0.9910	0.7171	1.7080	1.7181	99.4%	0.6%
9	1	2	0.0019	0.0003	0.0003	0.0005	0.0013	0.0013	0.0003	0.0003	NA	NA	0.0063	1.0067	0.8015	1.8081	1.8144	99.7%	0.3%
10	1	2	0.0011	0.0003	0.0002	0.0003	NA	NA	0.0000	0.0000	0.0059	0.0010	0.0088	0.6277	0.5486	1.1763	1.1851	99.3%	0.7%
11	1	2	0.0011	0.0004	0.0000	0.0004	0.0001	0.0003	0.0001	0.0001	0.0051	0.0006	0.0082	0.5490	0.4001	0.9491	0.9573	99.1%	0.9%
12	1	2	0.0809	0.0006	0.0005	0.0003	0.0003	0.0002	0.0001	0.0001	0.0045	0.0006	0.0880	1.0787	0.3652	1.4439	1.5319	94.3%	5.7%
13	2	1	0.0661	0.0035	0.0001	0.0012	0.0011	0.0020	0.0001	0.0002	0.0097	0.0014	0.0854	0.9929	1.5036	2.4965	2.5819	96.7%	3.3%
14	2	1	0.0316	0.0030	0.0002	0.0005	0.0006	0.0015	0.0001	0.0001	0.0153	0.0021	0.0550	1.4991	0.3893	1.8885	1.9435	97.2%	2.8%
15	2	1	0.0195	0.0032	0.0002	0.0020	0.0027	0.0029	0.0002	0.0002	0.0226	0.0036	0.0571	0.8202	0.4664	1.2865	1.3436	95.8%	4.2%
16	2	2	0.1658	0.0042	NA	0.0028	0.0005	0.0005	0.0001	0.0001	0.0019	0.0001	0.1759	0.1985	1.3191	1.5177	1.6936	89.6%	10.4%
17	2	2	0.1176	0.0303	0.0213	0.0291	0.0018	0.0016	0.0000	0.0001	0.0084	0.0013	0.2114	0.0198	0.6114	0.6312	0.8426	74.9%	25.1%
18	2	2	0.0808	0.0089	0.0043	0.0071	0.0014	0.0017	0.0001	0.0001	0.0119	0.0017	0.1181	0.5493	0.5944	1.1437	1.2618	90.6%	9.4%
19	3	1	0.0782	0.0036	0.0066	0.0012	0.0009	0.0015	0.0001	0.0000	0.0063	0.0020	0.1005	2.3439	1.1553	3.4992	3.5996	97.2%	2.8%
20	3	1	0.0522	0.0005	0.0001	NA	0.0000	NA	0.0000	0.0000	0.0006	0.0003	0.0536	2.0998	1.1007	3.2006	3.2542	98.4%	1.6%
21	3	1	0.2385	0.0028	0.0059	0.0007	0.0086	0.0063	0.0004	0.0005	0.0052	0.0025	0.2714	2.2860	0.7278	3.0138	3.2852	91.7%	8.3%
22	3	2	0.0349	0.0003	0.0000	NA	0.0000	NA	0.0000	0.0000	0.0002	0.0001	0.0356	1.6707	0.8041	2.4748	2.5104	98.6%	1.4%

[a] 1: Western fast food kitchen, 2: Chinese cafeteria kitchen, 3: Street food stand; [b] 1: male, 2: female; [c] No available information

Abbreviation: ACE = acenaphthene; ACY = acenaphthylene; ANT = anthracene; BaA = benzo(a)anthracene; CHR = chrysene; FLU = fluorene; FLUA = fluoranthene; FA = formaldehyde; AA = acetaldehyde

Table S5. Reproducibility, calibration line parameter, recovery rate, LOD, LOQ and MDL of 16 target PAHs.

Chemicals	RT	Conc. Range	Recovery rate (%)		Coefficient of variation for analysis	Calibration line parameter			MDL (μg/m³)		**TEF**
	(min)	(μg/mL)	Filter[a]	PUF	(%)	Slope	R^2	R	Filter	PUF	
Naphthalene	9.57	0 ~ 4.5	1.6%	47.4%	2.45%	0.298	0.9955	0.9978	0.164	0.007	0.001
Acenaphthylene	13.14	0 ~ 4.5	4.5%	53.4%	2.89%	0.867	0.9984	0.9992	0.025	0.003	0.001
Acenaphthene	13.58	0 ~ 4.5	12.3%	61.1%	4.93%	0.607	0.9975	0.9987	0.008	0.002	0.001
Fluorene	14.77	0 ~ 4.5	16.3%	66.5%	2.88%	0.606	0.9979	0.9989	0.008	0.003	0.001
Phenanthrene	17.16	0 ~ 4.5	29.7%	62.4%	3.37%	0.645	0.9994	0.9997	0.003	0.002	0.001
Anthracene	17.29	0 ~ 4.5	42.9%	66.6%	5.16%	0.460	0.9971	0.9985	0.003	0.003	0.01
Fluoranthene	21.07	0 ~ 4.5	50.1%	60.0%	8.58%	0.354	0.9977	0.9989	0.005	0.006	0.001
Pyrene	21.87	0 ~ 4.5	50.2%	58.0%	9.27%	0.351	0.9965	0.9982	0.005	0.005	0.001
Benzo(a)anthracene	26.82	0 ~ 4.5	83.7%	74.4%	4.02%	1.783	0.9986	0.9993	0.002	0.003	0.1
Chrysene	26.98	0 ~ 4.5	80.5%	64.1%	5.10%	1.883	0.9986	0.9993	0.005	0.008	0.01
Benzo(b)fluoranthene	31.35	0 ~ 4.5	80.9%	53.3%	8.21%	12.411	0.9966	0.9983	0.025	0.049	0.1
Benzo(k)fluoranthene	31.45	0 ~ 4.5	67.4%	53.1%	9.58%	13.012	0.9969	0.9985	0.026	0.042	0.1
Benzo(a)pyrene	32.56	0 ~ 4.5	85.6%	62.2%	10.27%	8.868	0.9982	0.9991	0.015	0.026	1
Indeno(1,2,3-cd)pyrene	36.68	0 ~ 4.5	101.1%	63.5%	15.98%	6.978	0.9970	0.9985	0.008	0.016	0.1
Dibenz(a,h)anthracene	36.78	0 ~ 4.5	73.4%	54.6%	15.85%	3.258	0.9985	0.9992	0.009	0.015	1
Benzo(g,h,i)perylene	37.51	0 ~ 4.5	86.6%	52.4%	13.16%	8.388	0.9976	0.9988	0.011	0.023	0.01

[a] 2-ring and 3-ring PAHs tend to be vaporized from filters; therefore, the recovery rates of these PAHs are low.

Table S6. Reproducibility, calibration line parameter, recovery rate, LOD, LOQ and MDL of 13 target aldehydes.

Chemical	RT	Conc. Range	Recovery rate (%)		Coefficient of variation for analysis	Calibration line parameter			LOD	LOQ	MDL (μg/m^3)	
	(min)	(μg/mL)	Filter	Cartridge	(%)	Slope	R^2	R	(μg/mL)		Filter	Cartridge
Formaldehyde	13.91	0~0.3	53.7%	73.8%	1.2%	1115486	0.9991	0.9995	0.003	0.008	0.078	0.113
Acetaldehyde	17.15	0~0.3	50.8%	59.9%	0.9%	702825	0.9994	0.9997	0.003	0.012	0.112	0.189
Acrolein	21.15	0~0.3	48.0%	46.1%	0.9%	882754	0.9995	0.9997	0.006	0.021	0.121	0.252
Propinoaldehyde	22.18	0~0.3	48.0%	46.1%	1.3%	629733	0.9995	0.9997	0.006	0.023	0.142	0.297
Crotonaldehyde	25.04	0~0.3	53.5%	52.5%	2.3%	688393	0.9984	0.9992	0.015	0.045	0.200	0.408
Butyraldehyde	26.56	0~0.3	53.5%	52.5%	1.1%	508931	0.9994	0.9997	0.010	0.034	0.160	0.326
Valeraldehyde	31.27	0~0.3	51.1%	67.0%	1.8%	421727	0.9993	0.9997	0.013	0.044	0.279	0.426
Hexaldehyde	36.85	0~0.3	65.4%	71.7%	2.3%	365895	0.9994	0.9997	0.008	0.028	0.154	0.281
t-2-Heptenal	40.14	0~0.32	97.0%	82.8%	2.1%	271439	0.9984	0.9992	0.017	0.060	0.220	0.515
t,t-2,4-Nonadienal	45.41	0~0.32	99.0%	95.7%	3.6%	132043	0.9989	0.9995	0.038	0.121	0.380	0.786
t-2-Nonenal	48.05	0~0.32	98.0%	84.3%	2.7%	273351	0.9997	0.9999	0.012	0.046	0.158	0.367
t,t-2,4-Decadienal	48.79	0~0.32	93.2%	93.8%	2.9%	129284	0.9986	0.9993	0.026	0.083	0.324	0.644
Nonanal	49.23	0~0.32	86.7%	95.1%	2.2%	179631	0.9995	0.9997	0.025	0.088	0.250	0.455
